# Policies and Initiatives Aimed at Addressing Research Misconduct in High-Income Countries

**DOI:** 10.1371/journal.pmed.1001406

**Published:** 2013-03-26

**Authors:** David B. Resnik, Zubin Master

**Affiliations:** 1 National Institute of Environmental Health Sciences, National Institutes of Health, Research Triangle Park, North Carolina, United States of America; 2 Alden March Bioethics Institute, Albany Medical College, Albany, New York, United States of America

## Abstract

David Resnik and Zubin Master review current policies and initiatives for preventing and managing research misconduct in high-income countries, summarize some high profile cases of misconduct, and make suggestions on ways forward.

**Summary Points**

- Most high-income countries have developed policies and initiatives to address research misconduct, including regulations, ethical guidance, professional standards, journal policies, education in the responsible conduct of research, and oversight by national bodies and research institutions.
- Some high-income countries have not developed policies and initiatives, and oversight of research integrity in these countries continues to evolve.
- Since misconduct is a global concern, international guidelines, such as the Singapore Statement on Research Integrity, are an important step toward international cooperation on research integrity.

The article by Joseph Ana and colleagues in this issue of *PLOS Medicine* provides valuable information that addresses key issues about research misconduct in low- and middle-income countries (LMICs) [1]. The authors show that while misconduct is a significant concern in these countries, most have done little to address the problem at a national level, with the notable exception of China. Many high-income countries (HICs) have developed policies and initiatives to address misconduct. Governance of research integrity in each nation usually involves a patchwork of instruments that may include a national policy, a central governing body, state/provincial standards, international standards, journal policies, professional guidelines, and institutional policies and oversight. At the national level, different countries have adopted a range of policy instruments and governance mechanisms to preserve and oversee research integrity. Policies can be written as legislation and regulations (e.g., United States, Denmark), guidelines (e.g., Canada), or professional standards and may be overseen by a governmental or non-governmental organization with a range of oversight functions. Although most HICs have developed comprehensive national policies, it is important to realize that many have not, and that oversight of research integrity in HICs continues to evolve. In this commentary, we will discuss a few exemplary policies and governance models in HICs, and make some suggestions to move forward.

## United States

Misconduct emerged as an issue in the US in the mid-1980s, when congressional committees held hearings on fraud and conflict of interest (COI) in federally funded research. One of the most well-known cases involved Nobel Prize–winning scientist David Baltimore, who was a professor of biology at the Massachusetts Institute of Technology (MIT) and directed the Whitehead Institute, which was operated by MIT and Tufts University. Baltimore was a coauthor on a paper with five other authors published in *Cell* in 1986, which came into question. Shortly after the paper was published, a post-doctoral researcher in Theresa Imanishi-Kari's laboratory, Margot O'Toole, had trouble repeating some of the experiments reported in the paper that were conducted by Imanishi-Kari. O'Toole asked to look at Imanishi-Kari's notebooks. When she could not reconcile data contained in the notebooks with the data reported in the paper, she accused Imanishi-Kari of misconduct. After an investigation by Tufts and MIT found no misconduct, the Office of Research Integrity (ORI) and congressional committee investigated the case. ORI found that Imanishi-Kari had committed misconduct by fabricating and falsifying data, but this ruling was overturned by a federal appeals panel in 1996, 10 years after the publication of the paper. Though Baltimore was never implicated in the scandal, his career and reputation were damaged. Imanishi-Kari has maintained that she did nothing wrong, except poor recordkeeping [2]. In response to this scandal and others, federal granting agencies, such as the National Institutes of Health (NIH) and National Science Foundation (NSF) began developing policies addressing misconduct and COI [3]. After more than a decade of debate in which agencies used different definitions of misconduct, the federal government agreed upon a common definition of research misconduct, which is defined as fabrication, falsification, or plagiarism (FFP). Misconduct does not include honest error or differences of scientific opinion [4]. This definition is narrower than a definition that had been used by some agencies, which included FFP as well as other serious deviations from accepted scientific practice. This latter category was rejected on the grounds that it was judged to be so vague and all-encompassing that it is difficult to enforce. Federal agencies also agreed upon procedures for investigating misconduct, which promote fairness and due process and protect the rights of the accused as well as those of whistle blowers [3].

The US also developed a system for overseeing integrity in federally funded research. Institutions are required to investigate allegations of misconduct for their federally funded research and report the results of their investigations to federal agencies, which review the investigations and make their own findings. Penalties imposed by federal agencies may include a ban on receiving federal research funding, and institutional penalties range from a reprimand to termination of employment. The ORI oversees research integrity in studies funded by the Public Health Service, which includes NIH-funded research. The Office of Inspector General at the NSF oversees research integrity in NSF-funded research [5].

Federal agencies also recognize that addressing misconduct involves much more than developing and enforcing rules: steps also must be taken to prevent misconduct. Education in the responsible conduct of research (RCR) is the primary means of preventing misconduct. Since 1989, the NIH has required instruction in RCR for graduate students supported by NIH funds. This requirement has evolved over the years to include post-doctoral students, fellows, researchers receiving special awards, and intramural researchers [6],[7]. The NIH requires that institutions provide instruction in eight specific content areas, and strongly encourages in-person training [5]. In 2009, the NSF implemented a congressional mandate for RCR education by requiring that institutions provide RCR instruction for undergraduate students, graduate students, and post-doctoral researchers supported by NSF funds [8]. The NSF does not impose specific training requirements on institutions but requires them to develop an RCR instructional plan [8]. More than half of US universities have gone beyond these federal mandates and require categories of individuals, such as all doctoral level students, all graduate students, or all researchers receiving external funding, to receive RCR education [9]. Universities have also developed their own research integrity policies, which cover not only misconduct, but also other areas of concern, such as COI, research record keeping, and intellectual property [5].

Federal agencies have also sponsored empirical and conceptual research, conferences, workshops, and course development related to research integrity. Federally funded research projects have covered such topics as the prevalence, incidence, and causes of unethical behavior in science; commercialization of research and COI; and openness and sharing of scientific data and materials [10].

One of the most important questions in research on research integrity concerns the effectiveness of RCR education. A meta-analysis of 26 studies that evaluated the impact of different RCR instructional programs found that education can influence ethical knowledge, awareness, and reasoning [11]. A controlled trial that compared a group of students receiving RCR education to one that did not also found that education can enhance ethical knowledge, awareness, and reasoning [12]. No studies to date have shown that RCR education actually reduces the incidence of misconduct, while there is some evidence to question the effectiveness of RCR education [13]. Clearly, more research on the effectiveness of different RCR educational methods and mentoring is needed.

## Canada

Canada has also had its share of misconduct scandals, which have stirred media interest and increased public awareness about research integrity. The most well-known case came to the surface in 1993, when Roger Poisson, a professor of surgery at the University of Montreal, admitted to fabricating and falsifying data for patients enrolled in the National Surgical Adjuvant Breast and Bowel Project (NSABP) between 1977 and 1990. Poisson altered data in order to ensure that his patients would qualify for the study. NSABP investigators reanalyzed the data after removing Poisson's patients from the study and found that the misconduct had no impact on the overall results. Nevertheless, the Poisson case undermined the public's trust in research and helped to spur reforms in Canada's research ethics policies [14].

In Canada, the conduct of research governed at the federal level is overseen by the three federal granting agencies collectively referred to as the Tri-Agencies: Natural Sciences and Engineering Research Council, Social Sciences and Humanities Research Council, and the Canadian Institutes of Health Research. Institutions receiving funds from the Tri-Agencies must comply with the Memorandum of Understanding (MOU), which outlines the responsibilities of individuals, institutions and the Tri-Agencies. Although there are several policies governing research under the MOU, the *Tri-Agency Framework: Responsible Conduct of Research* addresses research integrity. “Misconduct” is defined broadly under the Tri-Agency Framework, which contains provisions to maintain good recordkeeping, promote ethical authorship and publication practices, and manage COIs. The Framework also outlines policy breaches, including FFP, redundant publications, and invalid authorship and acknowledgement among others [15],[16]. Canada now requires that funded researchers disclose their personal information if a serious breach of Tri-Agency policies has been found [17]. Although the recently revised framework will serve to strengthen research integrity in Canada, further evidence-based research on research integrity in the Canadian context is sorely needed in order to understand the prevalence of misbehavior and continuously improve RCR practices [18].

## United Kingdom

Government funding for scientific research in the UK is provided by its seven Research Councils. The Research Council UK (RCUK) is an organization that oversees the different councils. Funding recipients must comply with a code of ethics established by the RCUK as well as ethical standards required by individual councils. The RCUK's code defines six areas of unacceptable research conduct (i.e., misconduct), including FFP, misrepresentation, mismanagement or inadequate preservation of data and/or primary materials, and breach of duty of care, which includes failing to take due care to protect human or animal subjects or the environment from harm. Institutions that receive funding from one of the Research Councils are responsible for publishing standards of conduct and investigating unacceptable behavior [19]. Additionally, medical researchers registered with the General Medical Council must abide by its guidance on research conduct. Failure to do so can bring their registration and license to practice medicine into jeopardy [20].

The UK Research Integrity Office (UKRIO) is an independent body and registered charity that provides expert advice and guidance about the conduct of research. Although it has no formal legal powers to investigate cases or administer punishments, the UKRIO provides confidential consultation on cases of alleged misconduct and publishes guidance on good research practices, misconduct investigation, and scientific retractions. The UKRIO also sponsors education and training on research integrity and publishes a blog and a list of useful resources on its website [21].

In April 2012, Universities UK, the Higher Education Funding Council for England, Research Councils UK, the Wellcome Trust, and several government departments drafted an agreement designed to promote integrity in UK research [22]. The agreement states that researchers, institutions, and sponsors should be committed to maintaining the highest standards of integrity in research. They should ensure that research is conducted according to ethical, professional, and legal standards; create a research environment founded on a culture of integrity, which includes policy development and a senior staff member responsible for coordinating research integrity efforts at the research institution; develop fair and transparent processes to deal with allegations of research misconduct; and regularly review efforts to promote research integrity [23].

## Denmark

In 1992, the Danish Medical Research Council established the Danish Committee on Scientific Dishonesty, which contained seven scientific members and was chaired by a high court judge [24]. The committee handled allegations of scientific misconduct and promoted RCR practices. At the time, the committee chose the term “scientific dishonesty” to reflect a broad range of misbehaviors beyond research fraud. In 1998, the Danish Research Councils created three committees together called the Danish Committees on Scientific Dishonesty (DCSD), which covers all fields of scientific research, including the natural and applied sciences, and the social sciences and humanities.

In 2001, Bjørn Lomborg, an adjunct professor at Copenhagen Business School, published *The Skeptical Environmentalist*, a book that challenged the consensus among climate scientists that global warming will lead to dire social and economic consequences [25]. In 2003, several scientists accused Lomborg of scientific dishonesty, alleging that he had fabricated, misrepresented, and misinterpreted data in the book. The DCSD found that he had committed scientific dishonesty, but its ruling was overturned by the Ministry of Science, Technology and Innovation, which said that the DCSD did not have sufficient evidence or arguments to support its ruling, and that the definition of dishonest was too vague [26]. As a result of the Lomborg case, new regulations were developed in 2005 that limited the scope of scientific dishonesty to FFP and other serious violations of good scientific practice committed intentionally or through gross negligence [27].

## European Code of Conduct for Research Integrity

The European Code of Conduct for Research Integrity (the Code) was the result of discussions by the European Science Foundation (ESF) Member Forum, and the Standing Committee on Science and Ethics of the All European Academies [28]. The Code received general approval from the European National Academies and the ESF Member Forum. The Code takes a comprehensive view of science including the natural and social sciences, and humanities. It outlines principles of scientific integrity and the responsibilities of researchers and institutions. The Code defines scientific integrity and summarizes misbehaviors including FFP, poor data management practices, improper research design, inappropriate authorship, poor publication and editorial practices, and insufficient respect and care of participants involved in research [29]. The Code also describes major and minor misdemeanors, taking into account researcher experience and the repetition of errors. Although the Code identifies unacceptable research practices, it contains positive elements and provides provisions on good scientific practice. The Code provides ethical guidance and does not supersede national laws.

## Institutional Oversight

While national policies can play an important role in the oversight of research integrity, the primary responsibility for oversight rests with research institutions. Research institutions are responsible for handling misconduct allegations, protecting whistleblowers from reprisal, developing and publicizing research integrity policies, and providing education on RCR. National policies and agencies can support institutional efforts, but they cannot take the place of robust institutional mechanisms and committed leadership [5]. For this reason, most national policies emphasize the importance of institutional oversight and responsibility. Although universities are best equipped to deal with misconduct within their jurisdiction, they may have COIs when it comes to investigating and reporting misbehaviors, because they may want to avoid a loss funding or harms to their reputation. Thus, national bodies free from COIs should oversee university efforts at promoting research integrity.

## Scientific Journals

Scientific journals also play a key role in addressing misconduct. About half of scientific journals have developed policies for responding to misconduct [30],[31]. Journals with higher impact factors are more likely to have such policies than journals with lower impact factors [32]. The Committee on Publication Ethics (COPE), a group of over 8,000 journal editors, publishers, and others interested in publication ethics, has developed publication ethics policies that have been adopted by journals that are members of COPE. The policies provide guidance for editors and reviewers, and address such matters as responding to allegations of misconduct in submitted or published articles, and correcting the publication record when misconduct has been confirmed. The COPE guidelines also recommend that journals have strategies in place to detect plagiarism, redundant publications, and inappropriate image manipulation [33].

## Professional Associations

Finally, numerous professional associations, including the American Anthropological Association, American Chemical Society, the American Physical Society, the American Society for Microbiology, the American Statistical Association, the European Federation of Psychologist's Associations, the International Association of Synthetic Biology, the International Federation of Consulting Engineers, the International Society for Environmental Epidemiology, and the World Nuclear Association, have developed research ethics codes and policies that address research integrity issues. Professional societies can help to support an environment that fosters ethical conduct by establishing expected standards of behavior and providing guidance for scientists concerning ethical dilemmas and problems [34].

## Conclusion

Although HICs have developed different mechanisms for overseeing research integrity, much more work is needed. HICs that have not developed national laws or guidelines should do so. Further research should be conducted on the effectiveness of different national strategies for promoting and overseeing research integrity. Finally, it is important to recognize that research integrity is not just an issue for HICs or LMICs; it is a global concern. Scientists, government agencies, universities, scientific journals, and professional associations from different countries should work together to promote ethical conduct in research and address misconduct [35].

Since scientists from different countries may have different understandings of the concepts related to research integrity, it is important to develop international guidelines. One such attempt is the Singapore Statement on Research Integrity, developed by 341 individuals from 51 countries at the 2nd World Conference on Research Integrity in 2010. International policies are an important step toward international cooperation on research ethics and integrity issues, but additional efforts are needed [36].

## Abbreviations

COPE: Committee on Publication Ethics
FFF: fabrication, falsification, or plagiarism
HIC: high-income country
MIT: Massachusetts Institute of Technology
NIH: National Institutes of Health
NSF: National Science Foundation
ORI: Office of Research Integrity
RCR: responsible conduct of research
UKIRO: UK Research Integrity Office

## References

[1] Ana J, Koehlmoos T, Smith R, Yan LL (2013) Research misconduct in low- and middle-income countries. PLoS Med 10: e1001315 doi:10.1371/journal.pmed.1001315 23555197 10.1371/journal.pmed.1001315 PMC3608538

[2] Kevles D (1998) The Baltimore case: a trial of politics, science, and character. New York: W.W. Norton. 10.1136/bmj.319.7214.926 PMC1116746 10506071

[3] Steneck NH (1999) Confronting misconduct in science in the 1980s and 1990s: what has and has not been accomplished? Sci Eng Ethics 5: 161–176. 11657853 10.1007/s11948-999-0005-x

[4] Office of Science and Technology Policy (2000) Federal Research Misconduct Policy. Federal Register, 6 December 2000 65 (235) 76260–76264.

[5] Shamoo AS, Resnik DB (2009) Responsible conduct of research. 2nd ed. New York: Oxford University Press.

[6] National Institutes of Health (2011) Update on the requirement for instruction in responsible conduct of research. Available: http://grants1.nih.gov/grants/guide/notice-files/NOT-OD-10-019.html. Accessed 1 November 2012.

[7] National Institutes of Health (2000) NIH policy on instruction in the responsible conduct of research. Available: http://sourcebook.od.nih.gov/ResEthicsCases/nih-policy.htm. Accessed 1 November 2012.

[8] National Science Foundation (2009) Responsible conduct of research. Federal Register, 20 August 2009 74 (160) 42126–42128.

[9] Resnik DB, Dinse GE (2012) Do U.S. research institutions meet or exceed federal mandates for instruction in responsible conduct of research? A national survey. Acad Med 87: 1237–1242. 22836835 10.1097/ACM.0b013e318260fe5c PMC3430795

[10] Office of Research Integrity (2012) RRI Publications. Available: http://ori.hhs.gov/rri-publications. Accessed 1 November 2012.

[11] Antes AL, Murphy ST, Waples EP, Mumford MD, Brown RP, et al (2009) A meta-analysis of ethics instruction effectiveness in the sciences. Ethics Behav 19: 379–402. 19838311 10.1080/10508420903035380 PMC2762211

[12] May DR, Luth MT (2012) The effectiveness of ethics education: a quasi-experimental field study. Sci Eng Ethics Epub ahead of print 3 Jan 2012. 10.1007/s11948-011-9349-0 22212360

[13] Risbey KR, Ronning EA, De Vries R, Martinson BC (2007) What do mentoring and training in the responsible conduct of research have to do with scientists' misbehavior? Findings from a national survey of NIH-funded scientists. Acad Med 82: 853–860. 17726390 10.1097/ACM.0b013e31812f764c

[14] Angell M, Kassirer JP (1994) Setting the record straight in the breast cancer trials. N Engl J Med 330: 1448–1450. 8159201 10.1056/NEJM199405193302010

[15] Tri-Agencies (2011) The Tri-Agency Framework: responsible conduct of research. Available: http://www.rcr.ethics.gc.ca/eng/policy-politique/framework-cadre/. Accessed 6 November 2012.

[16] Master Z (2012) The governance of research integrity in Canada. Health Law Rev 20: 5–14.

[17] Tri-Agencies (2011) Agency statement: consent to disclose personal information. Available: http://www.nserc-crsng.gc.ca/NSERC-CRSNG/governance-gouvernance/consent-consentement_eng.asp. Accessed 6 November 2012.

[18] Master Z, McDonald M, Williams-Jones B (2012) Promoting research on research integrity in Canada. Account Res 19: 47–52. 22268504 10.1080/08989621.2012.638597

[19] UK Research Council (2012) The research ethics guidebook. Available: http://www.ethicsguidebook.ac.uk/Research-Council-funding-122. Accessed 2 November 2012.

[20] General Medical Council. Research guidance. Available: http://www.gmc-uk.org/guidance/ethical_guidance/research.asp. Accessed 18 December 2012.

[21] UK Research Integrity Office (2012) About us. Available: http://www.ukrio.org/about-us/. Accessed 2 November 2012.

[22] Torjesen I (2012) Research bodies try to strengthen the integrity of UK research. BMJ 344: e2677. 22496343 10.1136/bmj.e2677

[23] Universities UK. The concordat to support research integrity, signed July 2012.

[24] Andersen D (2000) From case management to prevention of scientific honesty in Denmark. Sci Eng Ethics 6: 25–34. 11273432 10.1007/s11948-000-0019-x

[25] Lomborg B (2001) The skeptical environmentalist. Cambridge: Cambridge University Press.

[26] Editors of The New Atlantis (2004) The ideological environmentalist. The New Atlantis 4 (Winter): 108–110.

[27] The Danish Committees on Scientific Dishonesty (2009) Guidelines for good scientific practice with special focus on health science, natural science and technical science. Available: http://en.fi.dk/publications/2009/the-danish-committees-on-scientific-guidelines-for-good-scientific-practice. Accessed 6 November 2012.

[28] Drenth PJD (2010) A European code of conduct for research integrity. Available: http://www.allea.org/Content/ALLEA/Scientific%20Integrity/A%20European%20Code%20of%20Conduct%20for%20Research%20Integrity_final.10.10.pdf. Accessed 6 November 2012.

[29] European Science Foundation, All European Academies (2011) The European code of conduct for research integrity. Available: http://www.allea.org/Content/ALLEA/Scientific%20Integrity/Code_Conduct_ResearchIntegrity.pdf. Accessed 6 November 2012.

[30] Resnik DB, Peddada S, Brunson W Jr (2009) Research misconduct policies of scientific journals. Account Res 16: 2567. 10.1080/08989620903190299 PMC3943876 19757231

[31] Bosch X, Hernández C, Pericas JM, Doti P, Marušić A (2012) Misconduct policies in high-impact biomedical journals. PLoS ONE 7 (12) e51928 doi:10.1371/journal.pone.0051928 23284820 10.1371/journal.pone.0051928 PMC3526485

[32] Resnik DB, Patrone D, Peddada S (2010) Research misconduct policies of social science journals and impact factor. Account Res 17: 79–84. 20306350 10.1080/08989621003641181 PMC3065865

[33] Committee on Publication Ethics (2011) Code of conduct and best practice guidelines for journal editors. Available: http://publicationethics.org/files/Code_of_conduct_for_journal_editors_Mar11.pdf. Accessed 2 November 2012.

[34] Macrina FL (2007) Scientific societies and promotion of the responsible conduct of research: codes, policies, and education. Acad Med 82: 865–869. 17726393 10.1097/ACM.0b013e31812f7e58

[35] Resnik DB (2009) International standards for research integrity: an idea whose time has come? Account Res 16: 218–228. 20183162 10.1080/08989620903065350 PMC3965194

[36] Singapore Statement on Research Integrity (2010) Available: http://www.singaporestatement.org/. Accessed 2 November 2012.

